# A band of bound states in the continuum induced by disorder

**DOI:** 10.1038/s41598-018-23576-z

**Published:** 2018-03-26

**Authors:** Yi-Xin Xiao, Zhao-Qing Zhang, C. T. Chan

**Affiliations:** Department of Physics, Hong Kong University of Science and Technology, Hong Kong, China

## Abstract

Bound states in the continuum usually refer to the phenomenon of a single or a few discrete bound states embedded in a continuous spectrum of extended states. Here we propose a simple mechanism to achieve a band of bound states in the continuum in a class of disordered quasi-1D and quasi-2D systems, where the bound states and extended states overlap completely in a spectral range. The systems are partially disordered in a way that a band of extended states always exists, not affected by the randomness, whereas the states in all other bands become localized and cover the entire spectrum of extended states. We demonstrate such disordered-induced bound states in the continuum in disordered multi-chain and multi-layer systems.

## Introduction

Disorder plays an important role in many fields of physics. It is well known that interference between multiply scattered waves can disrupt the transport of electrons and classical waves in disordered media and make the transport diffusive^1,2^. In 1958, Anderson predicted that disorder can even completely stop diffusion^3^. A widely-accepted feature of Anderson localization is that all states are localized in one- (1D) and two-dimensional (2D) disordered systems due to coherent backscattering effects^1,2,4^, while three-dimensional (3D) random media have a mobility edge^1,2,5^ that separates localized states from extended states^6,7^. The above results hold in general for completely disordered systems in which localized states and extended states do not coexist. An interesting question is whether localized states can coexist with extended states if the system is not completely disordered. The answer is not obvious because any overlap of two kinds of states can result in resonance-type extended states even with an infinitesimal coupling.

A similar question on whether a bound state can exist in the continuum of extended states (BIC) had been raised by Von Neumann and Wigner in 1929^8^ shortly after the advent of quantum mechanics. In the past few years, BICs have been achieved by several different mechanisms in various ordered systems^9–25^. However, in those systems, only one or a few discrete BICs were achieved.

In this work, we propose a mechanism to achieve a band of BICs induced by disorder in a class of quasi-1D and -2D systems. The systems are partially disordered in a way that the Hilbert space can be partitioned into two subspaces, so that the states in one subspace are unaffected by the presence of randomness and are hence extended, whereas wavefunctions in all other bands get localized by randomness. Different from the BICs found previously, here the disorder-induced BICs form a band and spectrally overlap with the continuum of extended states. We explicitly demonstrate such bands of BICs in multi-chain and multi-layer systems.

**Figure 1:**
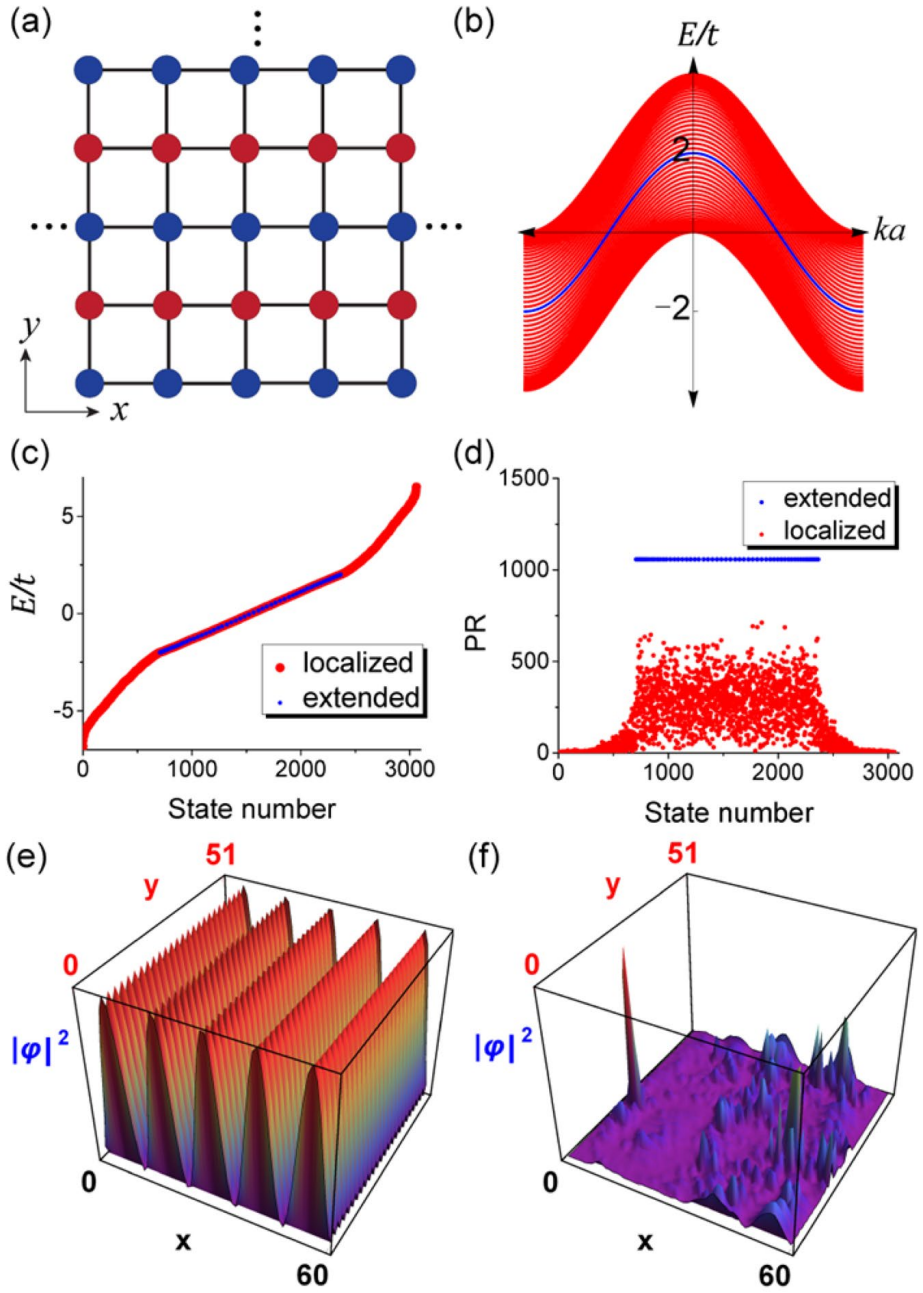
(**a**) A square lattice comprising multiple chains extending in the *x* direction. (**b**) The band structure with one band in blue color characterized by the same dispersion relation as that of an isolated chain. (**c**) The energy spectrum showing the spectral coexistence of extended states and localized states in the presence of onsite disorder on alternating chains (i.e. red sites). (**d**) The participation ratios of all the eigenstates. (**e**) and (**f**) show two states with the same eigen-energy *E*/*t* = 1.93, one is extended and the other is localized.

We first numerically demonstrate disorder-induced BICs in a multi-chain system described by a nearest-neighbor tight-binding Hamiltonian. The system consists of 2*N* + 1 (=51) coupled chains placed in the *x* direction, as depicted in Fig. 1(a). In the absence of disorder, the system is periodic in the *x* direction and all the chains are identical. There are 2*N* + 1 sites per unit cell. The lattice constant is *a* = 1, and all on-site energies and hopping parameters are taken to be zero and a constant *t*, respectively. The band structure of the multi-chain system, as shown in Fig. 1(b), comprises 2*N* + 1 bands. The central band marked by the blue curve has exactly the same dispersion relation $\,E/t=2\,\cos (ka)$ as that of a single chain, as we shall see later. Now we partially randomize the system by adding random on-site energies *ε*_*i*_ to every second chain, namely the red sites in Fig. 1(a). Now *N* out of 2*N* + 1 chains are disordered. To study the Anderson localization properties of such a system, we truncate each chain in the *x* direction to *M* (=60) sites (assuming hard-wall boundary condition) and numerically calculate the eigen-functions and the participation ratio (*PR*) of each eigenstate, which is defined as $PR=\frac{{({\sum }_{n}{|{c}_{n}|}^{2})}^{2}}{{\sum }_{n}{|{c}_{n}|}^{4}}$, where *c*_*n*_ are the components of an eigenstate |*φ*〉 = ∑_*n*_*c*_*n*_|*n*〉 and |*n*〉 denotes the atomic orbitals. For the case of uniform randomness *ε*_*i*_/*t* ∈ [−5, 5], the calculated energy spectrum and the participation ratios of all eigenstates are plotted in increasing energy in Fig. 1(c and d), respectively. Figure 1(d) shows clearly a stark contrast of two types of states: Type 1 contains *M* (=60) states with large values of *PR* (marked by blue dots) and Type 2 contains 2*N* × *M* (=3000) states with much smaller *PR* (marked by red dots). These two types of states overlap spectrally in the band of extended states. We will show analytically later that Type 1 states are extended states having exactly the same spectrum as that of an isolated chain, i.e., the blue curve in Fig. 1(b), unaffected by the randomness, and Type 2 states are Anderson localized states. We show the wavefunctions of two types of states at the same chosen energy in Fig. 1(e and f), which clearly demonstrate their spatial overlap. Thus, we have demonstrated Anderson localized states and extended states in a band region, namely a band of disorder-induced BICs in such systems. It should be stressed that although the extended states constitute an insignificant portion, i.e.1/(2*N* + 1), of the whole space of eigenstates in the thermodynamic limit *N* → ∞, nevertheless they occupy a significant portion of the entire spectrum as shown in Fig. 1(b and c).

The occurrence of extended states in such a partially disordered system is explained as follows. Basically, such systems consist of multiple identical copies of *A* subsystems with adjacent copies separated and coupled by an intermediate *B* subsystem. The *A* and *B* subsystems can be chains or layers. In the absence of randomness, *B* subsystems are also identical. A simple example has been shown in Fig. 1(a), in which *A* and *B* subsystems are identical chains but denoted by blue and red, respectively. The Hamiltonian of a system containing *N* + 1 copies of *A* and *N* copies of *B* can be expressed as

1 $${H}_{sys}=(\begin{array}{lllll}{H}_{A} & 0 & \cdots  & 0 & {T}_{1}^{\dagger }\\ 0 & {H}_{A} & \cdots  & 0 & {T}_{2}^{\dagger }\\ \vdots  & \vdots  & \ddots  & \vdots  & \vdots \\ 0 & 0 & \cdots  & {H}_{A} & {T}_{N+1}^{\dagger }\\ {T}_{1} & {T}_{2} & \cdots  & {T}_{N+1} & { {\mathcal H} }_{B}\end{array}),$$

2 $${ {\mathcal H} }_{B}=diag({H}_{B},{H}_{B},\cdots ,{H}_{B}),$$

where *H*_*A*_ and *H*_*B*_ are, respectively, the Hamiltonians of *A* and *B* subsystems. ${ {\mathcal H} }_{B}$ contains *N* copies of *H*_*B*_. If we assume that all subsystems are finite, each with *M* sites, the dimensions of *H*_*A*_ and *H*_*B*_ are *M* × *M*. The dimensions of *H*_*sys*_ and $\,{ {\mathcal H} }_{B}$ in Eq. (1) become (2*N* + 1)*M* × (2*N* + 1)*M* and *NM* × *NM*, respectively. The block matrices *T*_*i*_ denote the couplings between *A* and *B* and have dimensions *M* × *NM*. The explicit form of *H*_*sys*_ in Eq. (1) for small values of *N* and *M* can be found in Supplementary Information. It will be shown analytically below that the coupled system *H*_*sys*_ always contains a set of eigenvalues which are the same as those of an isolated *A* subsystem, similar to the blue band in Fig. 1(b). This implies that the Hamiltonian can be block diagonalized to contain one isolated block *H*_*A*_, namely

3 $${Q}^{-1}{H}_{sys}Q=(\begin{array}{cc}{H}_{A} & 0\\ 0 &  {\mathcal H} \text{'}\end{array}),$$

where *Q* is a unitary matrix and $\, {\mathcal H} \text{'}$ is the other block with dimensions 2*NM* × 2*NM*. From Eq. (3), it is easy to see that the Hilbert space of the system is partitioned into two subspaces. Now we introduce randomness into every *B* subsystem, which randomizes the block $\, {\mathcal H} \text{'}$. Since the subspace associated with *H*_*A*_ is not affected by the disorder, all eigenstates in this subspace are extended. The randomness in the other subspace represented by $\, {\mathcal H} \text{'}$ gives rise to localized states. It is shown in Supplemental Information that each extended eigenstate in the *H*_*A*_ subspace is a direct sum of all eigenvectors of the *N* + 1 *A* subsystems, and has vanishing amplitudes on the *B* subsystems. Since the localized states in $\, {\mathcal H} \text{'}$ subspace also involve atomic orbitals of the *A* subsystem, the spatial coexistence of two types of states naturally occur and form a band of BICs. The spectral overlap can always be achieved by adjusting the randomness. We thus have a very general mechanism to achieve a band of bound states in a class of quasi-1D and -2D partially disordered systems.

**Figure 2:**
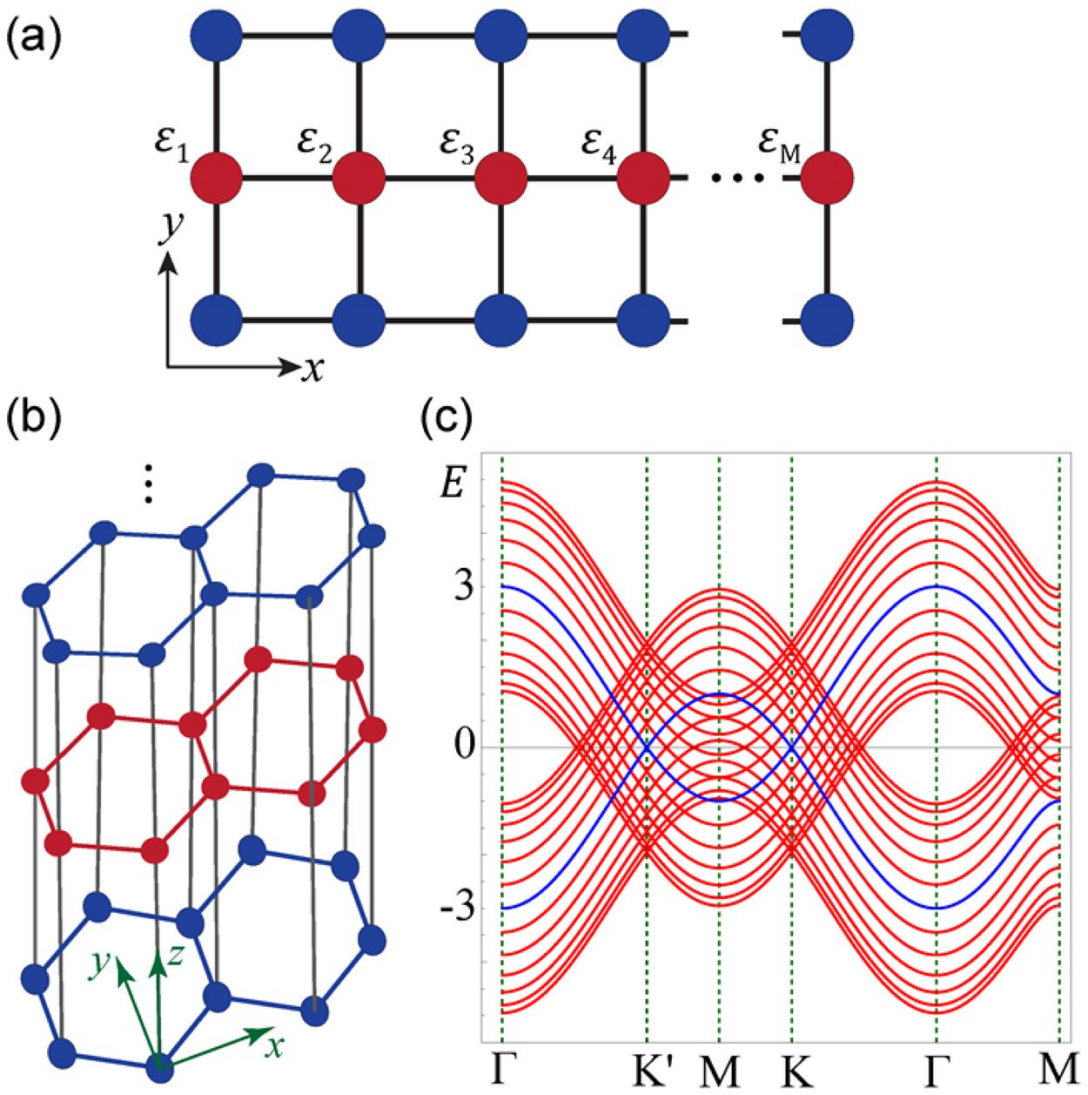
(**a**) A three-chain system: each chain contains *M* sites and on-site disorder is introduced to the middle chain. (**b**) A multilayer system comprising multiple AA-stacked honeycomb-lattice layers. (**c**) The band structure of the multilayer system.

To be more explicit, we consider a minimal model of three coupled chains, each truncated to contain *M* sites, where random on-site energies *ε*_*i*_ are introduced in the middle chain as shown in Fig. 2(a). The Hamiltonian of the system becomes

4 $${H}_{m}=(\begin{array}{ccc}{H}_{A} & 0 & {T}_{1}^{\dagger }\\ 0 & {H}_{A} & {T}_{2}^{\dagger }\\ {T}_{1} & {T}_{2} & {H}_{B}\end{array}),$$

where all the matrix elements are block matrices with dimensions *M* × *M*. *H*_*A*_ and *H*_*B*_ denote, respectively, the Hamiltonians of the blue and red chains. The inter-chain couplings are represented by *T*_1_ = *tI*_*M*_ and *T*_2_ = *tI*_*M*_, where *I*_*M*_ denotes an identity matrix. We assume that the single-chain Hamiltonian *H*_*A*_ satisfies the eigen-equation *H*_*A*_*P* = *P*Λ, where Λ = *diag*(*λ*_1_, *λ*_2_, …, *λ*_*M*_) is a diagonal matrix and *λ*_*i*_’*s* are the eigenvalues of *H*_*A*_, and *P* = (*φ*_1_, *φ*_2_, …, *φ*_*M*_) comprises *M* columns of eigenvectors of *H*_*A*_. A similarity transformation *H*_*S*_ = *X*^−1^ *H*_*m*_*X* with *X* = *diag*(*P*, *P*, *I*_*M*_) can be applied so that

5 $${H}_{S}=(\begin{array}{ccc}{\rm{\Lambda }} & 0 & {P}^{-1}{T}_{1}^{\dagger }\\ 0 & {\rm{\Lambda }} & {P}^{-1}{T}_{2}^{\dagger }\\ {T}_{1}P & {T}_{2}P & {H}_{B}\end{array}).$$

We note that when inter-chain couplings are absent, namely *T*_1_ = *T*_2_ = 0, there are two degenerate sets of eigenvalues *λ*_*i*_, *i* = 1, …, *M*. The presence of [*T*_1_*P*, *T*_2_*P*] can lift the degeneracy of *λ*_*i*_ but leave a set of *λ*_*i*_ as the eigenvalues of *H*_*s*_, thus also of *H*_*m*_, independent of *H*_*B*_. This can be seen as follows. For each degenerate pair of eigenvalue *λ*_*i*_ of *H*_*A*_, the effects due to *H*_*B*_ can be described by a simplified version of Eq. (5), i.e.,

6 $$(\begin{array}{ccc}{\lambda }_{i} & 0 & t{\phi }_{i}^{\dagger }\\ 0 & {\lambda }_{i} & t{\phi }_{i}^{\dagger }\\ t{\phi }_{i} & t{\phi }_{i} & {H}_{B}\end{array}).$$

Since the rank of the coupling block [*tφ*_*i*_, *tφ*_*i*_] is 1, where *φ*_*i*_ is the column eigenvector with respect to the eigenvalue *λ*_*i*_ of *H*_*A*_, by the rank-nullity theorem^26^, *λ*_*i*_ remains to be an eigenvalue of *H*_*S*_, thus also of *H*_*m*_, independent of *H*_*B*_. Applying the same argument to all *λ*_*i*_, we have shown that a set of eigenvalues *λ*_*i*_ of *H*_*A*_ remains and, therefore, *H*_*m*_ can always be block diagonalized into the following form,

7 $${H}_{BD}=(\begin{array}{cc}{H}_{A} & 0\\ 0 &  {\mathcal H} \text{'}\end{array})=(\begin{array}{ccc}{H}_{A} & 0 & 0\\ 0 & {H}_{A} & {R}^{\dagger }\\ 0 & R & {H}_{B}\end{array}),$$

by a unitary transformation *H*_*BD*_ = *Q*^−1^ *H*_*m*_*Q*. Concrete examples with explicit block diagonalization can be found in Supplementary Information. The above block diagonalization means a particular separation of Hilbert space into two subspaces. We call the subspace spanned by the eigenvectors corresponding to the invariant eigenvalues *λ*_*i*_ as the invariant subspace. For each eigenvalue *λ*_*i*_, the associated eigenvector Φ_*i*_ can be expressed as the direct sum of three parts corresponding to the three chains, namely

8 $${{\rm{\Phi }}}_{i}=-\frac{1}{\sqrt{2}}{\phi }_{1,i}^{A}\oplus \frac{1}{\sqrt{2}}{\phi }_{2,i}^{A}\oplus {\phi }^{B},$$

where $\,{\phi }_{1,i}^{A}$ and ${\phi }_{2,i}^{A}$ are the normalized eigenvectors of the two *A* (blue) chains and both correspond to the eigenvalue *λ*_*i*_. And *φ*^*B*^ is a zero vector with *M* components. That is to say, the anti-symmetric “combination” of the eigenvectors $\,{\phi }_{1,i}^{A}$ and $\,{\phi }_{2,i}^{A}$ of the two separate blue chains constitutes an eigenvector Φ_*i*_ for the whole coupled-chain system, which has odd parity, conforming to the mirror symmetry in the *y* direction, and vanishes at the sites in the middle chain. The fact that the degeneracy of *λ*_*i*_ (in the absence of inter-chain couplings) outnumbers the coupling channels brought by inter-chain couplings guarantees *λ*_*i*_ to be an eigenvalue for the coupled-chain system. Specifically, it is essential that blue chains (*H*_*A*_) outnumbers red chains (*H*_*B*_) by one to achieve a block diagonal form with one diagonal entry being *H*_*A*_, as shown in Eq. (3). Note such a block diagonalization is valid for more general configurations of inter-chain couplings, such as including next-nearest-neighbor hoppings, as shown in Supplemental Information. Since the invariant subspace does not involve the sites at the middle chain, the disorder (both diagonal and off-diagonal) introduced into the middle chain will not affect the invariant subspace. Consequently, states in the invariant subspace will remain extended, whereas all other eigenstates corresponding to the $\, {\mathcal H} \text{'}$ subspace become localized due to the presence of disorder. The spectral coexistence and therefore a band of BICs can always be achieved by adjusting the randomness. Since both subspaces involve the atomic orbitals at the A chains, the extended states and localized states will surely also coexist spatially.

We can generalize the system from three chains to 2*N* + 1 chains with *N* + 1 identical *A* chains separated and coupled by another *N B* chains. Following the similar procedure, it can be shown that the Hilbert space can be split into two subspaces with an invariant subspace spanned by *M* eigenvectors having the form $\,{{\rm{\Phi }}}_{i}=({\oplus }_{n=1}^{N+1}{c}_{n}{\phi }_{n,i}^{A})\oplus {\phi }^{B}$ (*i* = 1, …, *M*), each corresponds to the eigenvalue *λ*_*i*_ of the whole coupled-chain system. Here $\,{\phi }_{n,i}^{A}$ is the normalized eigenvector for the *n*-th *A* chain with eigenvalue *λ*_*i*_ and *φ*^*B*^ is a *NM*-component zero vector. The key is that the identical *A* chains outnumbers the *B* chains by 1 so that eigenvector components on each adjacent pair of *A* chains add destructively and vanish at the intermediate *B* chain, as detailed in Supplementary Information. There is always an invariant subspace and all the eigenstates therein are extended, whatever disorder is added on the *B* chains. The coexistence of extended states and localized states shown in Fig. 1 validates the above analysis.

**Figure 3:**
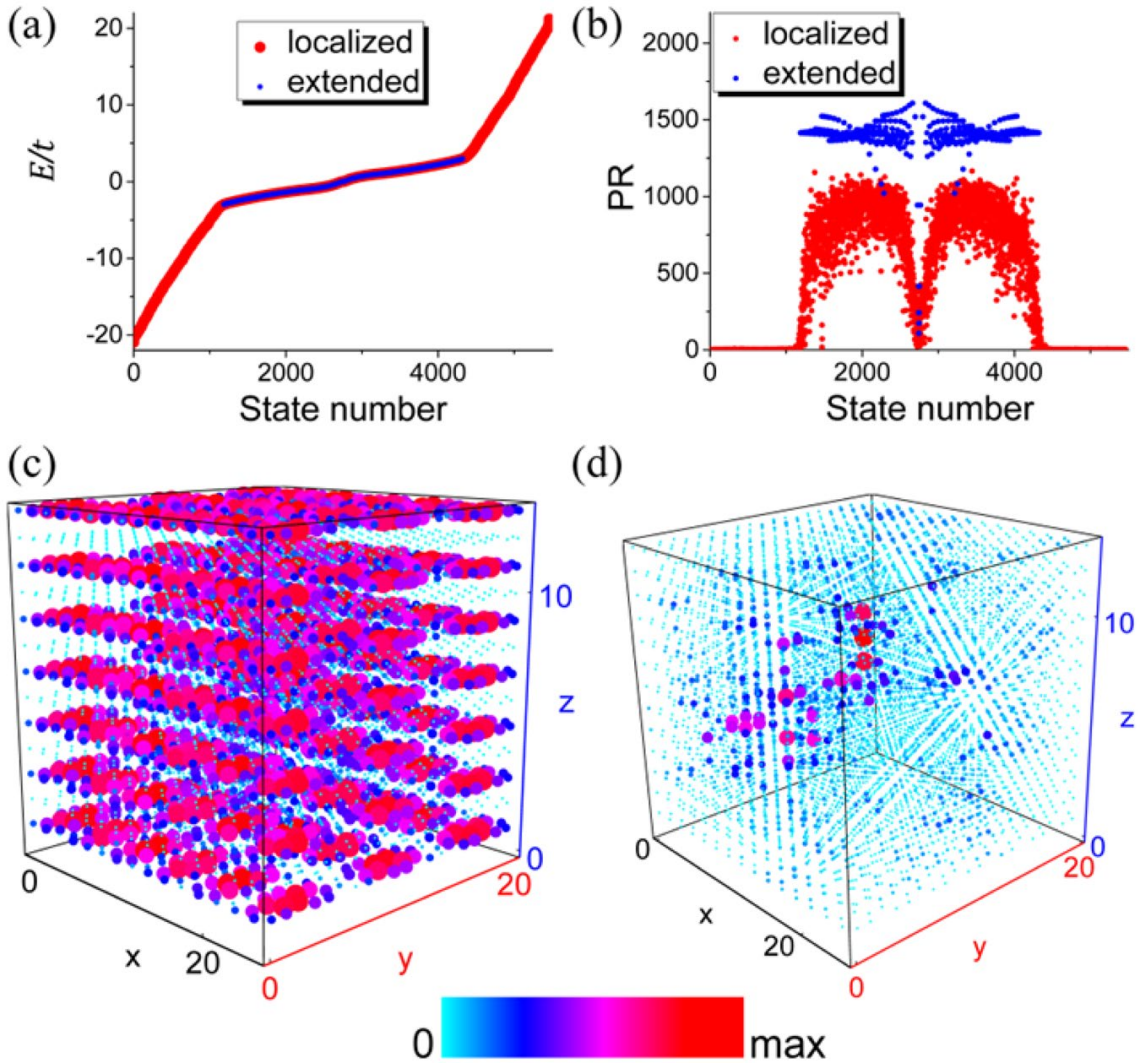
(**a**) The coexistence of extended states and localized states in the energy spectrum of a system consisting of multiple honeycomb layers, where diagonal disorder is added to alternating layers. (**b**) The participation ratios of all the eigenstates. (**c**) and (**d**) show two states with the same eigen-energy *E*/*t* = 2.51, one is extended and the other is localized.

Noticing the generality of the above analysis, it is quite obvious that the particular separation of Hilbert space is not limited to quasi-1D systems, but can also be applied to quasi-2D multilayer systems constructed similarly to achieve the spectral and spatial coexistence in coupled-layer systems. As an example, we consider a system comprising 2*N* + 1 (*N* = 6) AA-stacked honeycomb-lattice layers as shown in Fig. 2(b). Assuming the system is periodic in the *x* and *y* directions, we can compute its band structure. Since the honeycomb lattice is a two-band model, there should be totally 2(2*N* + 1) (=26) bands as shown in Fig. 2(c), in which two invariant bands are denoted by blue curves. We now introduce uniform diagonal disorder *ε*_*i*_ ∈ [−20, 20] to every *B* (red) layer and the system is truncated to contain 420 sites per layer. Open boundary conditions are assumed. The energy spectrum and participation ratios are shown in Fig. 3(a and b). They clearly show the spectral coexistence of the two different types of states in a band. A few eigenstates near zero energy have low participation ratios but are depicted by blue dots (marked as extended states) because they are edge states localized on the zigzag edges of ordered (*A*) layers and are actually from the invariant subspace. To further demonstrate the spectral coexistence, the absolute values of wavefunctions |*ψ*(*x*, *y*, *z*)| of two states at the same energy are denoted by colored dots at (*x*, *y*, *z*), as shown in Fig. 3(c and d). For better visualization, the sizes of the dots are scaled to be proportional to |*ψ*(*x*, *y*, *z*)|. The two states show markedly different nature: one is extended, and the other is localized. Their spatial coexistence is clearly seen.

In short, we proposed a method to create a band of disorder-induced BICs in a class of systems, in which the Hilbert space can be partitioned in a way that the disorder affects only one subspace causing localization, while the states in the other subspace remain extended. The disorder-induced BICs are demonstrated explicitly in both multi-chain and multi-layer systems. We want to emphasize that the method we propose is very general. It is not limited to the multi-chain and multi-layer systems demonstrated here. It applies universally to any similar structures as long as the identical copies of *A* subsystem outnumber that of *B* subsystem so that there is a particular subspace immune to degrees of freedom of the *B* subsystems, namely sites on the *B* subsystems. Such systems can be experimentally realized using coupled optical waveguides^27,28^ and cold atoms^29–32^. Our results imply that any energy stored in the localized states will not be carried away by energy transport in the eigen-channels of extended states although the energies in these two types of states overlap both spectrally and spatially.

## Electronic supplementary material

Supplementary information
