# Supplementary material for: A band of bound states in the continuum induced by disorder
Source: Sci Rep. 2018 Mar 26;8:5160. doi: 10.1038/s41598-018-23576-z (PMC5980084; doi:10.1038/s41598-018-23576-z)
Supplement: Supplementary file 1 — Supplementary information [file 41598_2018_23576_MOESM1_ESM.pdf]

Supplementary information for "A band of bound states in the continuum induced by disorder"

Yi-Xin Xiao,[1] Zhao-Qing Zhang,[1] and C. T. Chan[1]

[1]*Department of Physics, Hong Kong University of Science and Technology, Clear Water Bay, Kowloon, Hong Kong*

(Dated: January 1, 2018)

Abstract

In the main text, we have considered a class of systems, which consist of $N+1$ A subsystems and N B subsystems stacked in an alternating fashion, namely A-B-A-B-$\ldots$-A. The Hamiltonian of the system can be block diagonalized into two blocks so that one block is H_A, i.e., the Hamiltonian for an isolated A subsystem. The block diagonal representation implies that the Hilbert space is divided into two subspaces, each corresponding to one diagonal block. In this supplemental material, we analytically demonstrate that such a particular block diagonalization always exists in the aforementioned class of systems so that one block is H_A. And we give some concrete examples of the class of systems, and show explicitly the unitary transformation associated with the particular block diagonalization for the example systems. We also provide a general construction method to find the particular block diagonalization in such systems.

1. Explicit form of H_{sys}

We rewrite the Eq. (1) in the main text here,

$$H_{sys} = \begin{pmatrix} H_A & 0 & \cdots & 0 & T_1^\dagger \\ 0 & H_A & \cdots & 0 & T_2^\dagger \\ \vdots & \vdots & \ddots & \vdots & \vdots \\ 0 & 0 & \cdots & H_A & T_{N+1}^\dagger \\ T_1 & T_2 & \cdots & T_{N+1} & \mathcal{H}_B \end{pmatrix}, \tag{S1}$$

$$\mathcal{H}_B = diag(H_B, H_B, \cdots, H_B). \tag{S2}$$

We can write the Hamiltonian in Eq. (S1) explicitly as,

$$H_{sys} = \left(\begin{array}{ccccc|cccc} H_A & 0 & 0 & \cdots & 0 & T^\dagger & 0 & \cdots & 0 \\ 0 & H_A & 0 & \cdots & 0 & T^\dagger & T^\dagger & \cdots & 0 \\ 0 & 0 & H_A & \cdots & 0 & 0 & T^\dagger & \ddots & 0 \\ \vdots & \vdots & \vdots & \ddots & \vdots & \vdots & \vdots & \ddots & \ddots \\ 0 & 0 & 0 & 0 & H_A & 0 & 0 & \cdots & T^\dagger \\ \hline T & T & 0 & \cdots & 0 & H_B & 0 & \cdots & 0 \\ 0 & T & T & \cdots & 0 & 0 & H_B & \cdots & 0 \\ \vdots & \vdots & \ddots & \ddots & \vdots & \vdots & \vdots & \ddots & \vdots \\ 0 & 0 & 0 & \ddots & T & 0 & 0 & \cdots & H_B \end{array}\right), \tag{S3}$$

which contains $N+1$ subsystems A and N subsystems B. The block matrix T denotes the coupling between a subsystem A and a subsystem B, and has the expression $T = tI_M$, where I_M denotes an identity matrix.

2. Concrete examples: periodic systems and invariant bands

If we assume the system in Fig. 1 is infinite and periodic in the x direction, the block matrices H_A, H_B and T in Eq. (S3) reduce to three numbers, which have the following form in general:

$$H_A = \varepsilon_A + 2t_x \cos(ka), \quad H_B = \varepsilon_B + 2t_x \cos(ka), \quad T = t_y, \tag{S4}$$

where ε_A and ε_B denote on-site energies for sites on the A chains and B chains and k, respectively, denotes the Bloch wave vector. Here we use t_x to denote the hoppings in each chain along the x direction, and use t_y to denote the inter-chain hoppings in the y direction. t_x and t_y are not necessarily equal. Note we have implictly assumed that each isolated chain is a one-band system like in Fig. 1 (a); otherwise H_A (H_B) can be a $n \times n$ matrix if A (B) chain is n-band model, for example, the quasi-2D model in Fig. 3 in the main text comprises multiple honeycomb-lattice layers, with each layer being a two-band model.

If we consider the minimal system with only two identical A chains coupled by one B chain, namely $N = 1$, the Hamiltonian of the coupled-chain then becomes

$$H_{sys}^{N=1}(k) = \left(\begin{array}{cc|c} \varepsilon_A + 2t_x\cos(ka) & 0 & t_y \\ 0 & \varepsilon_A + 2t_x\cos(ka) & t_y \\ \hline t_y & t_y & \varepsilon_B + 2t_x\cos(ka) \end{array}\right), \tag{S5}$$

where k dependence of the Hamiltonian is shown explicitly. Obviously, such a system can be explicitly block diagonalized to

$$H_{BD}^{N=1}(k) = \left(\begin{array}{c|cc} \varepsilon_A + 2t_x\cos(ka) & 0 & 0 \\ \hline 0 & \varepsilon_A + 2t_x\cos(ka) & \sqrt{2}t_y \\ 0 & \sqrt{2}t_y & \varepsilon_B + 2t_x\cos(ka) \end{array}\right), \tag{S6}$$

by the unitary transformation $Q^{-1}H_{sys}^{N=1}(k)Q = H_{BD}^{N=1}(k)$, where

$$Q = \begin{pmatrix} -\frac{1}{\sqrt{2}} & \frac{1}{\sqrt{2}} & 0 \\ \frac{1}{\sqrt{2}} & \frac{1}{\sqrt{2}} & 0 \\ 0 & 0 & 1 \end{pmatrix}. \tag{S7}$$

In the absence of couplings between A and B chains, there are two degenerate bands $E(k) = \varepsilon_A + 2t_x\cos(ka)$. The presence of the inter-chain couplings t_y will split the degeneracy; however, there will always be one band with the dispersion relation $E(k) = \varepsilon_A + 2t_x\cos(ka)$ remaining for $H_{sys}^{N=1}(k)$ whatever nonzero value t_y takes. The eigenvector associated with the invariant band for $H_{sys}^{N=1}(k)$ is simply $\psi = [-1, 1, 0]^T$.

In the general case with $N + 1$ A chains and N B chains, the Hamiltonian $H_{sys}(k)$ takes

the form,

$$H_{sys}(k) = \left(\begin{array}{ccccc|cccc} \alpha_A(k) & 0 & 0 & \cdots & 0 & t_y & 0 & \cdots & 0 \\ 0 & \alpha_A(k) & 0 & \cdots & 0 & t_y & t_y & \cdots & 0 \\ 0 & 0 & \alpha_A(k) & \cdots & 0 & 0 & t_y & \ddots & 0 \\ \vdots & \vdots & \vdots & \ddots & \vdots & \vdots & \vdots & \ddots & \ddots \\ 0 & 0 & 0 & 0 & \alpha_A(k) & 0 & 0 & \cdots & t_y \\ \hline t_y & t_y & 0 & \cdots & 0 & \alpha_B(k) & 0 & \cdots & 0 \\ 0 & t_y & t_y & \cdots & 0 & 0 & \alpha_B(k) & \cdots & 0 \\ \vdots & \vdots & \ddots & \ddots & \vdots & \vdots & \vdots & \ddots & \vdots \\ 0 & 0 & 0 & \ddots & t_y & 0 & 0 & \cdots & \alpha_B(k) \end{array}\right), \tag{S8}$$

where $\alpha_A(k) = \varepsilon_A + 2t_x \cos(ka)$ and $\alpha_B(k) = \varepsilon_B + 2t_x \cos(ka)$. We can similarly use the rank-nullity theorem argument [1] to show that there is one band with $E(k) = \alpha_A(k) = \varepsilon_A + 2t_x \cos(ka)$ associated with $H_{sys}(k)$. We denote the coupling block at the left-bottom of $H_{sys}(k)$ as H_{AB}. It has dimensions of $N \times (N+1)$ and obviously has a full rank; therefore, $rank(H_{AB}) = N$. So the nullity, namely the dimension of the null space, of H_{AB} is $nul(H_{AB}) = N + 1 - rank(H_{AB}) = 1$, due to the rank-nullity theorem [1]. That is to say, there is one but only one vector ζ which satisfies $H_{AB}\zeta = 0$. And it is easy to find that $\zeta = [-1, 1, -1, 1, \cdots, (-1)^{N+1}]^T$, which contains $N+1$ components. Immediately, we notice that $\psi = \zeta \oplus \chi = [-1, 1, -1, 1, \cdots, (-1)^{N+1}, 0, 0, \cdots, 0]^T$, where χ is a N-component zero vector, constitutes an eigenvector of $H_{sys}(k)$, of which the corresponding eigenvalue is $E(k) = \alpha_A(k)$. Hence, one band of the couplec-chain system has exactly the same dispersion relation $E(k) = \varepsilon_A + 2t_x \cos(ka)$ as that of a single isolated chain. Thus we have explained why there is a blue band with the exactly same dispersion relation as that of a single isolated chain in Fig. 1(b) in the main text. Obviously, the argument also applies to coupled-layer systems consisting of layers described by one-band models.

In a more general system with each isolated chain or layer being a n-band model, a similarity transformation is needed to arrive at the form like in Eq. (S8), as discussed for a finite system in the main text. Then there are n bands which have the same dispersion relations as those of a single isolated n-band chain (layer), as demonstrated in Fig. 2 (b) in the main text.

In the main text, we mentioned that the particular block diagonalization is also valid for configurations including next-nearest-neighbor hoppings. We consider a example system as

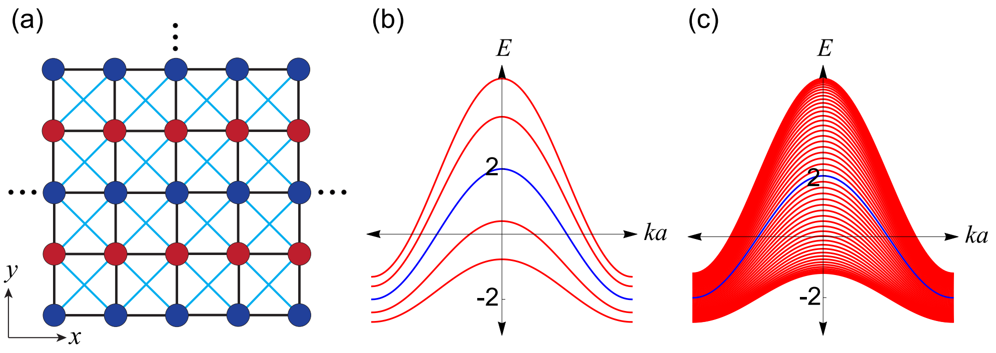

FIG. S1. (a) shows a coupled-chain system comprising $N+1$ A (blue) chains and N B (red) chains. Next-nearest-neighbor hoppings denoted by blue lines are included. (b) The band structure for $N = 2$ system. (c) The band structure for $N = 25$ system. The NN and NNN hoppings are assumed to be $t_1 = 1$ and $t_2 = 0.3$, respectively.

shown in Fig. S1(a).

We denote nearest-neighbor (NN) and next-nearest-neighbor (NNN) hoppings as t_1 and t_2, respectively. If $N = 2$, namely there are three A (blue) chains and two B (red) chains, the Bloch Hamiltonian has the following form:

$$H_{NNN}(k) = \begin{pmatrix} \alpha_A(k) & T(k) & 0 & 0 & 0 \\ T(k) & \alpha_B(k) & T(k) & 0 & 0 \\ 0 & T(k) & \alpha_A(k) & T(k) & 0 \\ 0 & 0 & T(k) & \alpha_B(k) & T(k) \\ 0 & 0 & 0 & T(k) & \alpha_A(k) \end{pmatrix}, \tag{S9}$$

where $\alpha_A(k) = \varepsilon_A + 2t_1\cos(ka)$, $\alpha_B(k) = \varepsilon_B + 2\cos(ka)$ and $T(k) = t_1 + 2t_2\cos(ka)$. We notice that $\phi = [1, 0, -1, 0, 1]$ is an eigenstate of $H_{NNN}(k)$ corresponding to the eigenvalue $E = \alpha_A(k) = \varepsilon_A + 2\cos(ka)$. Likewise, we can also use the rank-nullity theorem to show that there is one invariant band $E = \alpha_A(k)$ for a general N. We first treat the $N = 2$ case. The above Hamiltonian $H_{NNN}(k)$ can be rearranged to the following form:

$$H'_{NNN}(k) = \left(\begin{array}{ccc|cc} \alpha_A(k) & 0 & 0 & T(k) & 0 \\ 0 & \alpha_A(k) & 0 & T(k) & T(k) \\ 0 & 0 & \alpha_A(k) & 0 & T(k) \\ \hline T(k) & T(k) & 0 & \alpha_B(k) & 0 \\ 0 & T(k) & T(k) & 0 & \alpha_B(k) \end{array}\right). \tag{S10}$$

We immediately have

$$rank(H_{AB}) = rank \begin{pmatrix} T(k) & T(k) & 0 \\ 0 & T(k) & T(k) \end{pmatrix} = 2, \tag{S11}$$

where the H_{AB} denotes the 2×3 coupling block. Therefore, the nullilty of the coupling block is $nul(H_{AB}) = N_{col} - rank(H_{AB}) = 3 - 2 = 1$, where N_{col} is the number of columns of the coupling block H_{AB}. The null vector of the coupling block is $\zeta = [1, -1, 1]^T$, namely, $H_{AB}\zeta = 0$. Since the the top-left block matrix $diag[\alpha_A(k), \alpha_A(k), \alpha_A(k)]$ is an identity matrix, $\psi = \zeta \oplus [0, 0]^T = [1, -1, 1, 0, 0]^T$ is its eigenvector corresponding to $E = \alpha_A(k)$, i.e., $H'_{NNN}(k)\psi = \alpha_A(k)\psi$. Note ψ here is equivalent to ϕ up to a re-arrangement of chains. We have shown that there is an invariant band $E = \alpha_A(k)$ for the $N = 2$ system. It is staightforward to observe that an invariant band must exist for a general system with $2N + 1$ chains using similar argument. We show the band structures for two different situations respectively in Fig. S1 (b) and (c): (b) $N = 2$ and (c) $N = 25$. For both cases, one band marked by a blue curve has exactly the same dispersion relation $E = \alpha_A(k)$ as that of a single chain. Therefore, the invariant band in the NNN case is identical to that in the NN case, since $\alpha_A(k) = \varepsilon_A + 2t_1 \cos(ka)$ does not depend on NNN hoppings t_2.

3. Concrete examples: finite systems with invariant eigen-energies

It now become transparent that similar block diagonalization surely exists in a finite system without assuming the periodic boundary condition. Therefore, in the presence of couplings, a set of eigen-energies rather than a set of bands will remain the same as those of an isolated finite chain or layer. In the following, we show a few specific examples. The minimal model with $N = 1$ shown in Fig. S2 is discussed in the main text, with the Hamiltonian H_m shown in Eq. (4). The similarity transformation given in Eq. (5) and the discussions followed have showed that H_m can be block diagonalized to contain one decoupled block H_A, which is presented in Eq. (7). All the discussions have been made based on an abstract matrix form, where the matrix entries are not explicitly shown. Here we show the explicit form of the Hamiltonian and its block diagonalization.

Example in Fig. S2(a): Without loss of generality, we assume that each chain contains only $M = 2$ sites for simplicity, which is the system shown in Fig. S2(a).

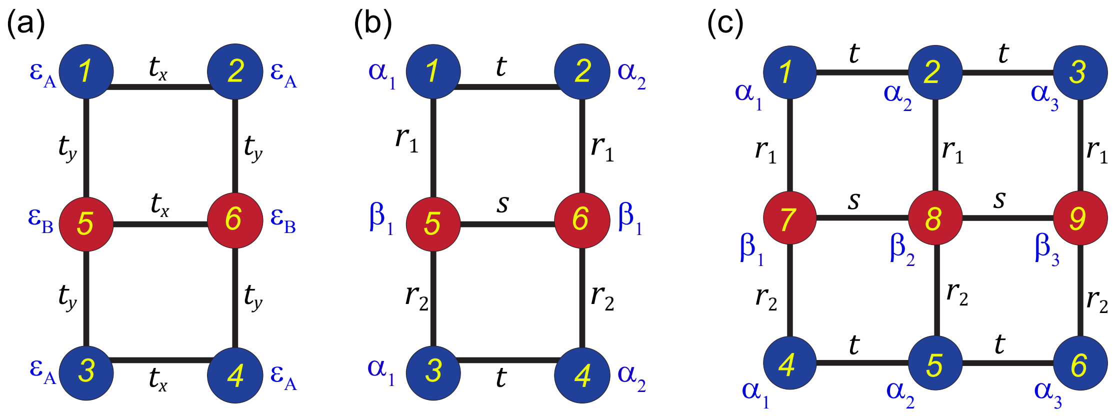

FIG. S2. (a) shows a small coupled-chain system comprising 2 A chains and 1 B chain, each containing 2 sites. (b) includes one more site on each chain. The on-site energies, hopping constants and atomic orbitals are labeled in both (a) and (b).

The Hamiltonian has the following form:

$$H_m = \left(\begin{array}{cc|c} H_A & 0 & T_1^\dagger \\ 0 & H_A & T_2^\dagger \\ \hline T_1 & T_2 & H_B \end{array}\right) = \left(\begin{array}{cccc|cc} \varepsilon_A & t_x & 0 & 0 & t_y & 0 \\ t_x & \varepsilon_A & 0 & 0 & 0 & t_y \\ 0 & 0 & \varepsilon_A & t_x & t_y & 0 \\ 0 & 0 & t_x & \varepsilon_A & 0 & t_y \\ \hline t_y & 0 & t_y & 0 & \varepsilon_B & t_x \\ 0 & t_y & 0 & t_y & t_x & \varepsilon_B \end{array}\right), \tag{S12}$$

where t_x denotes the hoppings along chains and t_y denotes inter-chain couplings. H_A, H_B, T_1 and T_2 are all 2×2 matrices here since $M = 2$. Now we look for the explicit form of the similarity transformation $H_S = X^{-1} H_m X$ which leads to Eq. (5). First we solve the eigen-equation $H_A \varphi = E\varphi$. We obtain two normalized eigenvectors

$$\varphi_1 = \begin{pmatrix} \frac{1}{\sqrt{2}} \\ \frac{1}{\sqrt{2}} \end{pmatrix}, \qquad \varphi_2 = \begin{pmatrix} -\frac{1}{\sqrt{2}} \\ \frac{1}{\sqrt{2}} \end{pmatrix}, \tag{S13}$$

which correspond to eigenvalues $\lambda_1 = \varepsilon_A + t_x$ and $\lambda_2 = \varepsilon_A - t_x$, respectively. We can rewrite the eigen-equation in the form of $H_A P = P\Lambda$, where

$$P = \begin{pmatrix} \frac{1}{\sqrt{2}} & -\frac{1}{\sqrt{2}} \\ \frac{1}{\sqrt{2}} & \frac{1}{\sqrt{2}} \end{pmatrix}, \quad \Lambda = \begin{pmatrix} \lambda_1 & 0 \\ 0 & \lambda_2 \end{pmatrix} = \begin{pmatrix} \varepsilon_A + t_x & 0 \\ 0 & \varepsilon_A - t_x \end{pmatrix}. \tag{S14}$$

The orthogonal matrix X for the similarity transformation then has the following form,

$$X = \left(\begin{array}{c|c|c} P & 0 & 0 \\ \hline 0 & P & 0 \\ \hline 0 & 0 & I_2 \end{array}\right), = \left(\begin{array}{cc|cc|cc} \frac{1}{\sqrt{2}} & -\frac{1}{\sqrt{2}} & 0 & 0 & 0 & 0 \\ \frac{1}{\sqrt{2}} & \frac{1}{\sqrt{2}} & 0 & 0 & 0 & 0 \\ \hline 0 & 0 & \frac{1}{\sqrt{2}} & -\frac{1}{\sqrt{2}} & 0 & 0 \\ 0 & 0 & \frac{1}{\sqrt{2}} & \frac{1}{\sqrt{2}} & 0 & 0 \\ \hline 0 & 0 & 0 & 0 & 1 & 0 \\ 0 & 0 & 0 & 0 & 0 & 1 \end{array}\right), \tag{S15}$$

Consequently, H_S in Eq. (5) then has the following explict form,

$$H_S = \begin{pmatrix} \Lambda & 0 & P^{-1}T_1^\dagger \\ 0 & \Lambda & P^{-1}T_2^\dagger \\ T_1P & T_2P & H_B \end{pmatrix} = \left(\begin{array}{cccc|cc} \lambda_1 & 0 & 0 & 0 & t_y & t_y \\ 0 & \lambda_2 & 0 & 0 & -t_y & t_y \\ 0 & 0 & \lambda_1 & 0 & t_y & t_y \\ 0 & 0 & 0 & \lambda_2 & -t_y & t_y \\ \hline t_y & -t_y & t_y & -t_y & \varepsilon_B & t_x \\ t_y & t_y & t_y & t_y & t_x & \varepsilon_B \end{array}\right), \tag{S16}$$

After rearranging H_S, we obtain

$$H_S' = \left(\begin{array}{cccc|cc} \lambda_1 & 0 & 0 & 0 & t_y & t_y \\ 0 & \lambda_1 & 0 & 0 & t_y & t_y \\ 0 & 0 & \lambda_2 & 0 & -t_y & t_y \\ 0 & 0 & 0 & \lambda_2 & -t_y & t_y \\ \hline t_y & t_y & -t_y & -t_y & \varepsilon_B & t_x \\ t_y & t_y & t_y & t_y & t_x & \varepsilon_B \end{array}\right), \tag{S17}$$

For the eigenvalue λ_1, the coupling block is

$$\begin{pmatrix} t_y & t_y \\ t_y & t_y \end{pmatrix}, \tag{S18}$$

of which the rank is 1. From the rank-nullity theorem [1], we are guaranteed to have one eigenvalue λ_1. Similarly, λ_2 must also be an eigenvalue of H_S'. Obviously, the eigenvectors for H_S' in Eq. (S17) are $[1, -1, 0, 0, 0, 0,]^T$ and $[0, 0, 1, -1, 0, 0,]^T$ for λ_1 and λ_2. Therefore, we have explicitly shown that there are always a set of eigenvalues, namely λ_1 and λ_2, which remain the same as those of a single isolated A chain described by H_A.

Naturally, the Hamiltonian H_m can be block diagonalized to the particular form which contains one decoupled block H_A. For this simple system here, we can explicitly show the unitary transformation in $H_{BD} = Q^{-1} H_m Q$, where

$$H_{BD} = \left(\begin{array}{cc|cccc} \varepsilon_A & t_x & 0 & 0 & 0 & 0 \\ t_x & \varepsilon_A & 0 & 0 & 0 & 0 \\ \hline 0 & 0 & \varepsilon_A & t_x & \sqrt{2}t_y & 0 \\ 0 & 0 & t_x & \varepsilon_A & 0 & \sqrt{2}t_y \\ 0 & 0 & \sqrt{2}t_y & 0 & \varepsilon_B & t_x \\ 0 & 0 & 0 & \sqrt{2}t_y & t_x & \varepsilon_B \end{array}\right), \tag{S19}$$

and

$$Q = \begin{pmatrix} -\frac{1}{\sqrt{2}} & 0 & \frac{1}{\sqrt{2}} & 0 & 0 & 0 \\ 0 & -\frac{1}{\sqrt{2}} & 0 & \frac{1}{\sqrt{2}} & 0 & 0 \\ \frac{1}{\sqrt{2}} & 0 & \frac{1}{\sqrt{2}} & 0 & 0 & 0 \\ 0 & \frac{1}{\sqrt{2}} & 0 & \frac{1}{\sqrt{2}} & 0 & 0 \\ 0 & 0 & 0 & 0 & 1 & 0 \\ 0 & 0 & 0 & 0 & 0 & 1 \end{pmatrix}. \tag{S20}$$

Example in Fig. S2(b): Actually the system can be more general than the one in Eq. (S12). We consider the following Hamiltonian with a general form,

$$H_g = \left(\begin{array}{cc|c} H_A & 0 & T_1^\dagger \\ 0 & H_A & T_2^\dagger \\ \hline T_1 & T_2 & H_B \end{array}\right) = \left(\begin{array}{cccc|cc} \alpha_1 & t & 0 & 0 & r_1 & 0 \\ t & \alpha_2 & 0 & 0 & 0 & r_1 \\ 0 & 0 & \alpha_1 & t & r_2 & 0 \\ 0 & 0 & t & \alpha_2 & 0 & r_2 \\ \hline r_1 & 0 & r_2 & 0 & \beta_1 & s \\ 0 & r_1 & 0 & r_2 & s & \beta_2 \end{array}\right), \tag{S21}$$

for the system shown in Fig. S2(b). Following the previous discussion for H_m, it is easy to find the unitary transformation $Q^{-1} H_g Q = H_{g,BD}(k)$ which block diagonalizes H_g to

$$H_{g,BD} = \left(\begin{array}{cc|cccc} \alpha_1 & t & 0 & 0 & 0 & 0 \\ t & \alpha_2 & 0 & 0 & 0 & 0 \\ \hline 0 & 0 & \alpha_1 & t & R & 0 \\ 0 & 0 & t & \alpha_2 & 0 & R \\ 0 & 0 & R & 0 & \beta_1 & s \\ 0 & 0 & 0 & R & s & \beta_2 \end{array}\right) \tag{S22}$$

where $R=\sqrt{r_1^2+r_2^2}$, with the unitary matrix

$$Q=\frac{1}{R}\begin{pmatrix} -r_2 & 0 & r_1 & 0 & 0 & 0 \\ 0 & -r_2 & 0 & r_1 & 0 & 0 \\ r_1 & 0 & r_2 & 0 & 0 & 0 \\ 0 & r_1 & 0 & r_2 & 0 & 0 \\ 0 & 0 & 0 & 0 & R & 0 \\ 0 & 0 & 0 & 0 & 0 & R \end{pmatrix}. \tag{S23}$$

Basically, the unitary matrix Q is constructed based on the eigenstate of an isolated column of the system in the y direction with eigen-energy $E=\alpha_1$ ($E=\alpha_2$) if the 1st (2nd) column is chosen. Here the unitary matrix Q shows how the new basis underlying the $H_{g,BD}$ is expressed as linear combinations of the atomic orbitals of the system shown in Fig. S2(b), namely

$$\begin{aligned} |1'\rangle &= \frac{1}{R}[-r_2\,|1\rangle + r_1\,|3\rangle], \quad & |2'\rangle &= \frac{1}{R}[-r_2\,|2\rangle + r_1\,|4\rangle], \\ |3'\rangle &= \frac{1}{R}[r_1\,|1\rangle + r_2\,|3\rangle], \quad & |4'\rangle &= \frac{1}{R}[r_1\,|2\rangle + r_2\,|4\rangle], \\ |5'\rangle &= |5\rangle\,, & |6'\rangle &= |6\rangle\,. \end{aligned} \tag{S24}$$

The first two equations in (S24) show that the basis $|1'\rangle$ and $|2'\rangle$ underlying the H_A block in $H_{g,BD}$ is anti-symmetric "combinations" of atomic orbitals in the two chains.

Example in Fig. S2(c): Next we consider another concrete example shown in Fig. S2(c) with $M=3$. The Hamiltonian has the following form,

$$H^{N=1,M=3}=\left(\begin{array}{ccc|ccc|ccc} \alpha_1 & t & 0 & 0 & 0 & 0 & r_1 & 0 & 0 \\ t & \alpha_2 & t & 0 & 0 & 0 & 0 & r_1 & 0 \\ 0 & t & \alpha_3 & 0 & 0 & 0 & 0 & 0 & r_1 \\ \hline 0 & 0 & 0 & \alpha_1 & t & 0 & r_2 & 0 & 0 \\ 0 & 0 & 0 & t & \alpha_2 & t & 0 & r_2 & 0 \\ 0 & 0 & 0 & 0 & t & \alpha_3 & 0 & 0 & r_2 \\ \hline r_1 & 0 & 0 & r_2 & 0 & 0 & \beta_1 & s & 0 \\ 0 & r_1 & 0 & 0 & r_2 & 0 & s & \beta_2 & s \\ 0 & 0 & r_1 & 0 & 0 & r_2 & 0 & s & \beta_3 \end{array}\right). \tag{S25}$$

The Hamiltonian can be block diagonalized into the following form

$$H^{BD}(k) = \left(\begin{array}{ccc|cccccc} \alpha_1 & t & 0 & 0 & 0 & 0 & 0 & 0 & 0 \\ t & \alpha_2 & t & 0 & 0 & 0 & 0 & 0 & 0 \\ 0 & t & \alpha_3 & 0 & 0 & 0 & 0 & 0 & 0 \\ \hline 0 & 0 & 0 & \alpha_1 & t & 0 & R & 0 & 0 \\ 0 & 0 & 0 & t & \alpha_2 & t & 0 & R & 0 \\ 0 & 0 & 0 & 0 & t & \alpha_3 & 0 & 0 & R \\ 0 & 0 & 0 & R & 0 & 0 & \beta_1 & s & 0 \\ 0 & 0 & 0 & 0 & R & 0 & s & \beta_2 & s \\ 0 & 0 & 0 & 0 & 0 & R & 0 & s & \beta_3 \end{array}\right). \tag{S26}$$

by the unitary transformation $Q^{-1}H(k)Q = H^{BD}(k)$, where $R = \sqrt{r_1^2 + r_2^2}$ and

$$Q = \frac{1}{R}\begin{pmatrix} -r_2 & 0 & 0 & r_1 & 0 & 0 & 0 & 0 & 0 \\ 0 & -r_2 & 0 & 0 & r_1 & 0 & 0 & 0 & 0 \\ 0 & 0 & -r_2 & 0 & 0 & r_1 & 0 & 0 & 0 \\ r_1 & 0 & 0 & r_2 & 0 & 0 & 0 & 0 & 0 \\ 0 & r_1 & 0 & 0 & r_2 & 0 & 0 & 0 & 0 \\ 0 & 0 & r_1 & 0 & 0 & r_2 & 0 & 0 & 0 \\ 0 & 0 & 0 & 0 & 0 & 0 & R & 0 & 0 \\ 0 & 0 & 0 & 0 & 0 & 0 & 0 & R & 0 \\ 0 & 0 & 0 & 0 & 0 & 0 & 0 & 0 & R \end{pmatrix}. \tag{S27}$$

Similarly, for this system with $M = 3$, the unitary matrix Q gives the following expressions of the new basis for H^{BD} in terms of atomic orbitals:

$$\begin{aligned} |1'\rangle &= \frac{1}{R}[-r_2\,|1\rangle + r_1\,|4\rangle], & |2'\rangle &= \frac{1}{R}[-r_2\,|2\rangle + r_1\,|5\rangle], & |3'\rangle &= \frac{1}{R}[-r_2\,|3\rangle + r_1\,|6\rangle], \\ |4'\rangle &= \frac{1}{R}[r_1\,|1\rangle + r_2\,|4\rangle], & |5'\rangle &= \frac{1}{R}[r_1\,|2\rangle + r_2\,|5\rangle], & |6'\rangle &= \frac{1}{R}[r_1\,|3\rangle + r_2\,|6\rangle], \\ |7'\rangle &= |7\rangle\,, & |8'\rangle &= |8\rangle\,, & |9'\rangle &= |9\rangle\,. \end{aligned} \tag{S28}$$

4. General construction of the block diagonalization for a multi-chain system

We can construct the unitary matrix which block diagonalize a general multi-chain system, such as the one shown in Fig. 1 in the main text. We denote the j-th orbital in

the i-th A (B) chain as $|i,j\rangle_A$ ($|i,j\rangle_B$), where $i = 1,2,\cdots,N+1$ ($i = 1,2,\cdots,N$) and $j = 1,2,\cdots,M$. The subspace associated with the block H_A involve only $|i,j\rangle_A$, whereas the other subspace associated with the block $\mathcal{H}'$ involves both $|i,j\rangle_A$ and $|i,j\rangle_B$.

We first look at the subspace associated with the block H_A. From previous concrete examples, we already know that the M basis vectors associated with the H_A subspace can be simply expressed as

$$|j\rangle_A^{H_A} = \sum_{i=1}^{N+1} a_i\,|i,j\rangle_A\,, \quad j = 1,2,\cdots,M, \tag{S29}$$

where the superscript "H_A" in $|j\rangle_A^{H_A}$ denote the H_A subspace and the subscript "A" means that only the atomic orbitals in the A chains are involved. Since each $|j\rangle_A^{H_A}$ involves only the j-th atomic orbital in each chain, therefore, ${}_A^{H_A}\langle m\,|j\rangle_A^{H_A} = \delta_{m,j}$. We denote the Hamiltonian of the first column of the system as H_0, which has the following form:

$$H_0 = \left(\begin{array}{ccccc|cccc}
\alpha_1 & 0 & 0 & \cdots & 0 & r_1 & 0 & \cdots & 0 \\
0 & \alpha_1 & 0 & \cdots & 0 & r_2 & r_3 & \cdots & 0 \\
0 & 0 & \alpha_1 & \cdots & 0 & 0 & r_4 & \ddots & 0 \\
\vdots & \vdots & \vdots & \ddots & \vdots & \vdots & \vdots & \ddots & \ddots \\
0 & 0 & 0 & 0 & \alpha_1 & 0 & 0 & \cdots & r_{2N} \\
\hline
r_1 & r_2 & 0 & \cdots & 0 & \beta_1 & 0 & \cdots & 0 \\
0 & r_3 & r_4 & \cdots & 0 & 0 & \beta_1 & \cdots & 0 \\
\vdots & \vdots & \ddots & \ddots & \vdots & \vdots & \vdots & \ddots & \vdots \\
0 & 0 & 0 & \ddots & r_{2N} & 0 & 0 & \cdots & \beta_1
\end{array}\right). \tag{S30}$$

The coefficients a_i in Eq. (S29) are extracted from the components of the eigenvector ψ_0 of H_0 corresponding to the eigenvalue $E = \alpha_1$. Here $\psi_0 = (c_1^A, c_2^A, \cdots, c_{N+1}^A, 0, 0, \cdots, 0)$. With ψ_0 computed, we have $a_i = c_i^A$. Using (S29), we have obtained all the M basis vectors $|j\rangle_A^{H_A}$ as linear combinations of the atomic orbitals $|i,j\rangle_A$.

Next, we aim to find the $2NM$ basis vectors which underlies the $\mathcal{H}'$ subspace. Since $|i,j\rangle_A$ are orthogonal to $|i,j\rangle_B$, $2NM$ basis vectors for the $\mathcal{H}'$ subspace can naturally be partitioned into two parts: one part only involves $|i,j\rangle_A$ and the other only involves $|i,j\rangle_B$. We denote the basis vectors in the two parts by $|i,j\rangle_A^{\mathcal{H}'}$ and $|i,j\rangle_B^{\mathcal{H}'}$, respectively, where $\mathcal{H}'$ labels the subspace and A (B) marks the associated atomic orbitals. The basis vectors $|i,j\rangle_A^{\mathcal{H}'}$ should be orthogonal to the already-known M vectors $|j\rangle_A^{H_A}$ expressed in (S29) and

therefore can be achieved one by one by using the Gram-Schmidt process. Of course, there can be multiple choices in the Gram-Schmidt process, which lead to different block-diagonal forms of the Hamiltonian, namely H_{BD}. Because there are totally $(N+1)M$ orthogonal atomic orbitals $|i,j\rangle_A$ and the number of $|j\rangle_A^{H_A}$ is M , there must be NM basis vectors $|i,j\rangle_A^{\mathcal{H}'}$ where $i=1,\cdots,N,\ j=1,\cdots,M$. The number of $|i,j\rangle_B^{\mathcal{H}'}$, $i=1,\cdots,N,\ j=1,\cdots,M$ is also NM since there are in total NM atomic orbitals $|i,j\rangle_B$. We can simply write

$$|i,j\rangle_B^{\mathcal{H}'} = |i,j\rangle_B \tag{S31}$$

without linearly combining $|i,j\rangle_B$.

Now we have obatined all the $(2N+1)M$ basis vectors $|j\rangle_A^{H_A}$, $|i,j\rangle_A^{\mathcal{H}'}$ and $|i,j\rangle_B^{\mathcal{H}'}$, the number of which are M, NM, NM, respectively. We arrange them into a matrix form and get the unitary matrix

$$Q = \Big(\ |1\rangle_A^{H_A},\ \cdots,\ |M\rangle_A^{H_A},\ |1,1\rangle_A^{\mathcal{H}'},\ \cdots,\ |N,M\rangle_A^{\mathcal{H}'},\ |1,1\rangle_B^{\mathcal{H}'},\ \cdots,\ |N,M\rangle_B^{\mathcal{H}'} \Big), \tag{S32}$$

where all kets are column vectors and have dimensions of $(2N+1)M\times 1$ and Q has dimensions of $(2N+1)M\times(2N+1)M$.

Obviously, the above construction method also applies to the multi-layer system stacked in the A-B-A-B-$\ldots$-A fashion without any modifications.

[1] C. D. Meyer, *Matrix Analysis and Applied Linear Algebra* (SIAM, 2000).
